# Effects of feeding starch sugar by-products on in situ rumen disappearance rate, growth performance, and carcass characteristics of late finishing Hanwoo steers

**DOI:** 10.5713/ab.21.0126

**Published:** 2021-08-21

**Authors:** Yongjun Choi, Geetae Park, Hyokon Kang, Jiyeon Ahn, Eunchae Lee, Youngjun Na, Sangrak Lee

**Affiliations:** 1 Department of Animal Science and Technology, Konkuk University, Seoul 05029, Korea; 2 Animal Data Laboratory, Antller Inc., Seoul 05029, Korea

**Keywords:** Starch Sugar By-product, *In situ* Disappearance Rate, Performance, Carcass Characteristics, Hanwoo Steers

## Abstract

**Objective:**

The aim of this study was to determine the effects of feeding starch sugar by-products (SSBs) on *in situ* disappearance rate, performance, and carcass characteristics of Hanwoo steers in the late finishing stage.

**Methods:**

To determine the *in situ* disappearance rate, nylon bags filled with 5 g of SSB were inserted into the ventral sac of two cannulated Holsteins cows and incubated for 0, 2, 4, 8, 16, 24, and 48 h. A total of 30 Hanwoo steers were fed the experimental diets, which were basal diet (control) and 7% SSB on an as-fed basis (4.35% dry matter [DM]), formulated according to requirements of the Korean Feeding Standard for Hanwoo. The experiment was conducted over 80 days using a completely randomized block design.

**Results:**

Soluble fraction *a* of DM and organic matter (OM) was 44.20% and 64.60% DM, fraction *b* was 23.00% and 19.40% DM, and *c* values (the rate of degradation of fraction *b*) were 0.04 and 0.04/h, respectively. The effective degradability of DM at rumen solid outflow rates of 0.02, 0.05, and 0.08/h was 59.83, 54.75, and 52.16, respectively, and for OM was 77.78, 73.52, and 71.34, respectively. Initial and final body weight, average daily gain, DM intake, and gain:feed did not differ significantly between control and SSB groups during the entire experimental period. Carcass traits of Hanwoo steers with SSB supplementation were not significantly different between treatments except for dressing percentage, which was greater with SSB treatment. The content of saturated fatty acid (SFA) was greater and that of unsaturated fatty acids (UFA) was lower in the SSB group than in the control group. The ratio of UFA to SFA was significantly lower in the SSB group than in the control group.

**Conclusion:**

A total mixed ration containing less than 4.0% DM of SSBs can be used in Hanwoo steers without a decrease in productivity and carcass traits.

## INTRODUCTION

**Figure 1:**
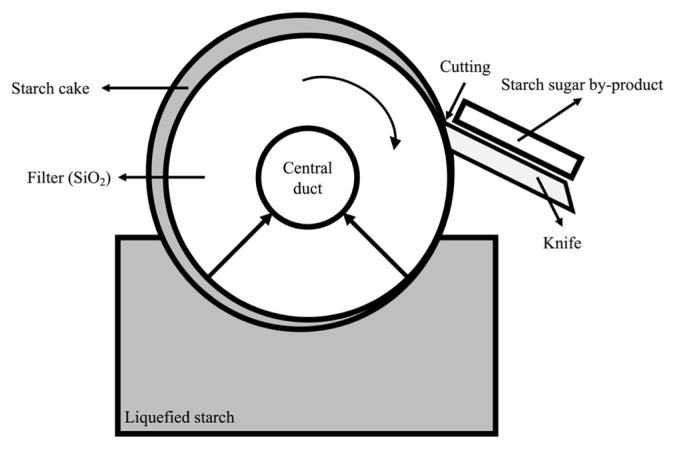
Starch sugar by-product (SSB) producing process. The target saccharides produced by fermentation are passed through the filter and remain stuck to the surface of the filter. The residues on the filter are peeled using a knife to maintain maximum absorption efficiency.

In the modern livestock industry, grains and pulses are used as common feed ingredients for high producing animals; however, there is competition for these ingredients with humans. A discarded agricultural by-product is a secondary product generated during the processing of grains, vegetables, or fruits [1]. Most by-products are human-inedible, and they can be utilized as an animal feed ingredient because they contain adequate organic materials for digestion. The starch sugar by-product (SSB) is produced by the following process: i) liquid starch passes through the filter to absorb useful saccharides into the center and ii) residue remains on the surface, which is separated using a knife [2] (Figure 1). The extracted sugar through the filter may contain starch in the residue and this could affect the rumen fermentation in case of use as a feed ingredient. Generally, because the filter is made of silica (SiO_2_), SSB indispensably contains not only starch sugar residue but also some of the silica which generated through the filter-cutting process. There was reported that the filter cake of sugarcane showed ash 10% to 14% dry matter (DM) and silica 4% to 10% DM of ash [3]. Although silica is an essential material for bone formation in young animals [4], a high level of silica in animal feed has a negative effect on the digestibility of cell wall components [5] and can induce formation of urinary tract stones (siliceous urinary calculi) [6]. Although a water-soluble form of silica in feed can have a negative effect on livestock, silica generally has a very low solubility (7 to 8 g per 50 kg BW in sheep) near rumen temperature [7]. However, since silica in SSB is not in a water-soluble form, the negative effect is small in the ruminant.

The market for grain-originated refined starches (glucose, oligosaccharide, or syrup) is increasing constantly, causing an increase in SSBs. Most starch and sugar are manufactured by six companies in South Korea and the SSB produced is about 1,000 tons per month in South Korea [8]. Generally, most SSB is immediately collected by waste treatment companies as soon as it is produced. In waste treatment companies, SSB has been sold as a feed ingredient when there is demand and otherwise discarded. These SSBs contain sufficient nutritive value to utilize as an animal feed ingredient [9]. However, some aspects need to be evaluated to eliminate potential anti-nutritional factors such as lipid and ash contents to affect the digestibility and toxicity in the ruminant. Currently, research on SSB effects on the ruminant is very scarce.

Therefore, the objectives of the present study were to evaluate the effect of SSBs on i) rumen *in situ* disappearance rate and ii) growth performance and carcass characteristics of late finishing Hanwoo steers.

## MATERIALS AND METHODS

### Animal care

All research protocols were approved by the Konkuk University Animal Care and Use Committee (approval number: KU18094).

### Starch sugar by-products

The SSB used in the experiment is the residue left after extracting starch sugar using corn as raw material, and it was obtained from the starch sugar factory of Sajo Donga Won Industry (Dangjin, Korea). The collected SSB sample was stored at −20°C until analysis and experimentation. The chemical composition of the SSBs is shown in Table 1 and Supplementary Table S1.

### *In situ* disappearance rate

The experiment was performed at an experimental farm at Chungju-si, Chungchungbuk-do, South Korea (latitude 37.07° and longitude 127.86°). Two ruminally cannulated Holstein Friesian cows were used. They were fed commercially prepared pellet feed (DM, 90.6%; crude protein [CP], 21.7% DM; neutral detergent fiber [NDF], 35.8% DM; acid detergent fiber [ADF], 14.1% DM; ether extract [EE], 5.2% DM; ash, 7.4% DM) and tall fescue (DM, 91.5%; CP, 7.7% DM; NDF, 68.7% DM; ADF, 40.5% DM; EE, 0.9% DM; ash, 7.9% DM) at a ratio of 7:3, respectively. The experimental diet was fed at 2% of body weight (BW) during the experimental periods and water and mineral blocks were provided *ad libitum*.

The dried SSB samples were milled to pass through a 1 mm screen (Wiley Mill; Thomas Scientific, Swedesboro, NJ, USA). Nylon bags (5×10 cm, 45 μm pore size; R510, ANKOM Inc., Macedon, NY, USA), in triplicate, filled with 5 g of SSB were inserted into the rumen ventral sac of two cannulated Holstein cows and incubated for 0, 2, 4, 8, 16, 24, 48, and 72 h according to the NRC guideline [6]. After incubation, the nylon bags were removed from the rumen, washed under running tap water for 2 min and then washed in the cold rinse cycle (10 min) by hand, dried at 60°C for 48 h, and then weighed for the analysis of DM and CP. Degradation was calculated using the formula proposed by Ørskov and McDonald [10]:


$$\text{P}=a+b\;(1-{\text{e}}^{-\text{ct}}),$$

where P is the actual degradation after time t; *a* is the intercept of the degradation curve at time zero; *b* is the potential degradability of the component of the protein which will, in time, be degraded; *c* is the rate constant for the degradation of *b*; and *t* is time. Regression analysis was performed using SAS PROC REG (Version 9.4, SAS Institute Inc., Cary, NC, USA) for estimation of the fraction *b*.

The effective degradability (ED) of DM and CP was calculated using the following equation:


ED=a+(b×c)/(c+k),

where *k* is the estimated rate of outflow from the rumen and *a*, *b*, and *c* are the same parameters as described above. The ED was estimated as ED2, ED5, and ED8, assuming rumen solid outflow rates of 0.02, 0.05, and 0.08/h, which are representative of low, medium, and high feed intake, respectively [11].

### Animal and experimental design

A total of 30 Hanwoo steers (28.2±0.26 months old, 806.2± 28.2 kg BW) were used for the experiment. Animals were allocated according to ages and initial BW and then allotted to six sawdust-bedded pens (five heads/pen) and randomly fed the experimental diets (Table 2) during the entire experimental periods. The treatments were basal diet (control) and 7.0% of SSB on an as-fed basis (4.35% of DM), with the diet formulated according to the requirements of the Korean Feeding Standard for Hanwoo [12] (Table 2). The experiment was conducted using a randomized complete block design for 80 days. Experimental diets were fed once a day at 0800 h *ad libitum* in the form of total mixed ration with 5% refusals. Water and mineral blocks were also available *ad libitum*. The offered diets and refusals were measured daily for the calculation of DM intake (DMI), and BW was measured at day 0 and 80 (at 858±7.8 days old, respectively) from the start of the experiment. The DMI and BW were used to calculate average daily gain (ADG) and gain:feed. Experimental diets were sampled monthly and stored at −20°C for subsequent chemical analysis. After the end of the study, animals were slaughtered in the commercial slaughterhouse (Meat Processing Facility, Gyoungsangbuk-do, Korea) using a pistol equipped with an air compressor.

### Chemical analysis

All samples were dried in a drying oven (HB-503-LF; Hanbaek Scientific Technology, Bucheon, Korea) at 60°C for 48 h. DM (method No. 937.01), CP (method No. 990.03), EE (method No. 920.39), ash, and silica (method No. 920.08) were analyzed according to the methods of AOAC International [13]. The NDF (method No. 2002.04) and ADF (method No. 973.18) were analyzed using the ANKOM Fiber Analyzer (A200, ANKOM Inc., USA) according to method proposed by Mertens [14] and Van Soest et al [15], respectively. Acid detergent lignin was determined using a filter bag (F57, Ankom Inc., USA) and beaker according to method described by Möller [16]. The non-fiber carbohydrate content was calculated by subtracting the sum of CP, EE, ash, and NDF from 100.

### Carcass traits

To evaluate the carcass traits, all animals were transported to a commercial slaughterhouse (Meat Processing Facility, Korea) and slaughtered after 24 h of rest. At 24 h postmortem, carcass traits, including back fat thickness, dressing percentage, *longissimus* muscle (LM) area, carcass weight, marbling score, and quality grade were determined following the guidelines of the Animal Products Grading Service, Korea [17]. Approximately 300 g of LM tissue samples were collected from all steers and stored with vacuum packing at −80°C until subsequent fatty acid (FA) analyses.

### Fatty acid composition analysis

For FA analysis, the lipid material of the SSB samples was extracted using a methanol:chloroform (1:2, v/v) solvent [18] and then methylated [19]. After pretreatment, the supernatant was injected into a gas chromatograph (Agilent 6890, NY, USA) equipped with a flame ionization detector and a capillary column (30 m×0.25 mm×0.25 μm; No. 122-3232, Agilent, USA) operated with the oven at 50°C [20]. The inlet and detector temperatures were 180°C and 250°C, respectively. Helium was used as the carrier gas.

### Statistical analysis

Data were analyzed using a MIXED procedure of the SAS 9.4 software (SAS Inst. Inc., Cary, NC, USA) [21] as a completely randomized block design. The model was:


$${\text{Y}}_{\text{i}(\text{t})}=\mu +{\text{B}}_{\text{i}}+{\text{T}}_{\text{j}}+{\text{E}}_{\text{ij}(\text{t})},$$

where μ is the average value, B_i_ is block, T_j_ is treatment value, and E_ij(t)_ is the error value. The fixed effect was SSB, and random effect was initial BW of each pen. Statistical significance was compared between control and SSB treatment groups using the T-test option. Least square means between treatments were assessed using a pairwise comparison method. Statistical difference and tendency were accepted at a p-value less than 0.05 and 0.10, respectively. All means are presented as least square means.

## RESULTS AND DISCUSSION

Dry matter and organic matter (OM) degradation parameters, and the ED values of the SSB are presented in Table 3. Soluble fraction *a* of DM and OM was 44.2% and 64.6% DM, fraction *b* of DM and OM was 23.00% and 19.40% DM, and constant *c* and OM was 0.04 and 0.04, respectively. The ED2, ED5, and ED8 of DM were 59.83, 54.75, and 52.16, respectively and those of OM were 77.78, 73.52, and 71.34, respectively. The SSBs are separated from the mixture of starch sugar liquid and filter using physical methods such as a blade (Figure 1) and this process might influence the physical and chemical composition of the SSBs. In a previous experiment, the SSB collected from multiple time-points showed a high variation among physical and chemical characteristics [22]. This high variation in the SSB is one of the factors that could make it difficult to use as a feed ingredient. The DM, CP, EE, ash, NDF, and ADF content in the SSB used in the previous experiment was 61.4%, 14.4%, 23.26%, 32.6%, 35.7%, and 31.3% of DM, respectively [22]. As a high ash content, containing silica, could have negative effects when fed to ruminants [6,7], a low percentage of the SSB (3% of DM) was fed to dairy cows in the previous study [22]. However, the SSB used in this study showed lower ash content (13.0% of DM) than that in the previous study (32.6% of DM; [22]), approximately half on an as-dry basis. Therefore, it was considered that a larger amount could be used, and approximately 4.35% DM of SSB was fed to Hanwoo steers in this experiment. Furthermore, the silica content of SSB used in this experiment showed 12.14% of DM (Table 1), feeding 4.35% of DM SSB to a ruminant is equivalent to feeding 0.53% DM silica, and it is considered that could be fed without any negative effects. The SSB used in this experiment showed a lower range of ED values than obtained in the previous study (63.3% vs 70.5% [22]). Furthermore, the ED value of the SSB in this experiment was lower than the commonly observed ED value for corn grain and soybean [23,24]. However, it is noteworthy that the degradation parameter constant c of the SSB in this study (constant c, 0.04) was lower than that of the SSB in the previous study (constant c, 0.23) [22]. Considering degradation parameters, fraction b indicates the potential degradability of the component of DM or CP which will, in time, degrade and constant c value indicates the rate constant for the degradation of fraction *b* [10]. It has been reported that as constant c value becomes closer to zero, the digestibility of feed ingredients in the rumen increases [25,26]. Considering the result of the *in situ* disappearance evaluation, the difference observed between the SSB used in this and the previous study could imply that the nutritional value of SSBs can be increased by changing the starch sugar production process.

Body weight, ADG, DMI, and gain:feed with the SSB supplementation are presented in Table 4. In the feeding trial, a significant difference in the ADG, DMI, and gain:feed was not observed with an as-fed total mixed ration including 7.0% (as-fed basis) of SSB when compared to that with the control. In this experiment, the chemical composition of the SSB was similar to that of rice bran; most of the rice bran was replaced by the SSB in the experimental feed. Although a significant difference was not observed between the control and SSB treatment groups, the magnitude of ADG and gain:feed in the SSB treatment group was greater than in the control. Regarding carcass traits, there was no significant difference in most traits except for the dressing percentage (Table 5). It has been reported that a high positive correlation exists between live weight and dressing percentage [27], with a greater correlation in young calves [28]. In contrast, considering cows, it has been reported that the type of diet fed had no association with dressing percentage in Holstein cows [29]. Therefore, the significant difference in dressing percentage between control and SSB treatment groups (p< 0.05) can be considered to have been affected by the initial and final BW. Although there was insufficient evidence to suggest that SSB in the diet improves the dressing percentage of Hanwoo steers; it was clear that the SSB is a sufficient substitute for rice bran at 7.0% (as-fed basis).

In the LM FA profiles, there was no significant difference in each FA (Table 6). Regarding FA content, there was no significant difference between the control and SSB treatment groups. However, there was a significant difference in the ratio of UFA to SFA between the two groups (p<0.05). Diet and feeding time are more important determinants of meat fat content and FA composition than cattle breed type [30] and most intramuscular fat is synthesized during a late finishing period in ruminants [31]. Although this feeding experiment was of a short term of 80 days, this was sufficient to evaluate the effect of feed on intramuscular fat synthesis. It is well established that there is an increase in monounsaturated fatty acids and a decrease in SFA with increasing time on a grain-based diet [32]. In this study, SFA in LM was greater in the SSB treatment group than in the control (p<0.05), because the non-fiber carbohydrate content of the SSB was lower than that of rice bran. A previous study reported that as carcass quality grade in Hanwoo increased, the ratio of monounsaturated fatty acids to SFA also increased [33]. In this study, although the number of the highest quality grade (1^++^ grade) steers was more observed with the SSB group than in the control, it needs to be stressed that the lowest quality grade (2 grade) steers were greater in the SSB group than the control. In other words, the possibility that feeding a high level of SSBs could negatively affect the intramuscular fat synthesis of Hanwoo should be considered. In summary, SSBs are a worthwhile by-product to use as an energy and protein source in ruminants. However, it is suggested that feeding is not implemented at more than a 4.0% of dry basis in Hanwoo.

## IMPLICATIONS

Starch sugar by-products in the diet can be used for Hanwoo steers without adverse effects on body weight gain and carcass traits. Because starch sugar by-products have adequate crude protein and high gross energy content as a feed ingredient, it is suggested that a total mixed ration containing less than 4.0% dry matter of starch sugar by-products can be used in Hanwoo steers without a decrease in productivity and carcass traits.

## Figures and Tables

**Table 1:** Chemical composition of rice bran and starch sugar by-product (n = 3)

Items	Rice bran	SSB
DM (%)	88.19±0.3	62.15±0.8
CP (% DM)	15.41±0.8	15.29±0.4
EE (% DM)	19.22±0.8	24.68±0.2
Ash (% DM)	9.17±0.9	12.96±0.5
NDF (% DM)	28.43±3.1	33.36±0.2
ADF (% DM)	12.17±0.9	5.71±0.1
ADL (% DM)	NA	2.31±0.1
NFC (% DM)	27.76±4.2	13.71±0.8
SiO_2_ (% DM)	NA	12.14±0.12

SSB, starch sugar by-product; DM, dry matter; CP, crude protein; EE, ether extract; NDF, neutral detergent fiber; ADF, acid detergent fiber; ADL, acid detergent lignin; NFC, non-fiber carbohydrate; NA, not analyzed.

**Table 2:** Ingredients and chemical composition of the total mixed rations

Items	Control	SSB
Ingredient (as-fed basis, %)		
Corn	25.0	26.7
Commercial feed^1)^, pelleted	10.0	10.0
Commercial feed^2)^, pelleted	15.0	15.0
Rice bran	7.0	-
SSB	-	7.0
Molasses	5.0	5.0
Soybean meal	3.0	3.3
Soybean curd cake	10.0	10.0
Straw	8.0	8.0
Water	17.0	15.0
Total	100.0	100.0
Chemical composition		
DM (%)	65.31	65.21
CP (% DM)	13.05	13.10
EE (% DM)	4.75	4.94
CF (% DM)	9.45	9.00
Ash (% DM)	5.81	6.30
NDF (% DM)	25.10	23.43
ADF (% DM)	13.07	12.20

SSB, starch sugar by-product; DM, dry matter; CP, crude protein; EE, ether extract; CF, crude fiber; NDF, neutral detergent fiber; ADF, acid detergent fiber; TDN, total digestible nutrients.

1) DM, 86.96%; TDN, 70.78%; CP, 10.5%; CF, 6.6%; NDF, 23.6%; ADF, 11.93%; Ash, 7.09%; Ca, 0.94%; P, 0.36%.

2) DM, 88.08%; TDN, 70.15%; CP, 20.1%; CF, 7.18%; NDF, 28.79%; ADF, 13.28%; Ash, 8.36%; Ca, 1.03%; P, 0.51%.

**Table 3:** Changes in *in situ* disappearance rate of dry and organic matter of the starch sugar by-product in the rumen

Items	*In situ* disappearance rate of SSB (% DM)	

	DM	OM
Incubation time (h)		
0	45.83	60.48
2	58.39	67.35
4	59.20	68.00
8	64.14	72.64
16	65.10	73.65
24	68.27	76.74
48	67.20	75.41
72	77.21	83.96
Degradation parameter^1)^		
*a* (% DM)	44.20	64.60
*b* (% DM)	23.00	19.40
*c* (h^−1^)	0.04	0.04
ED^2)^ (% DM)		
ED 2	59.83	77.78
ED 5	54.75	73.52
ED 8	52.16	71.34

SSB, starch sugar by-product; DM, dry matter; OM, organic matter; ED, effective degradability.

1) *a*, the water-soluble fraction which is rapidly washed out of bags and assumed to be completely degradable; *b*, the slowly degradable fraction; *c*, the rate of degradation of fraction “*b*”.

2) Fractional rate of passage out of the rumen, wherein ED2, ED5, and ED8 are assumed as 0.02, 0.05, and 0.08/h, respectively.

**Table 4:** Effects of the dietary starch sugar by-product as a total mixed ration ingredient on body weight, average daily gain, dry matter intake, and gain:feed of Hanwoo steers

Items	Control	SSB	SEM	p-value
Initial BW (kg)	801.9	810.4	17.8	0.753
Final BW (kg)	841.5	865.7	17.6	0.408
ADG (kg/d)	0.50	0.690	0.09	0.226
DMI (kg/d)	9.1	9.0	0.41	0.688
Gain:feed	0.054	0.077	0.02	0.189

SSB, starch sugar by-product; SEM, standard error of the mean; BW, body weight; ADG, average daily gain; DMI, dry matter intake.

**Table 5:** Effects of the dietary starch sugar by-product as a total mixed ration ingredient on carcass traits of Hanwoo steers

Items	Control	SSB	SEM	p-value
Backfat thickness (mm)	18.43	18.53	1.29	0.963
Dressing (%)	61.0	63.6	0.44	0.021
LM area (cm^2^)	103.68	102.11	1.53	0.507
Carcass weight (kg)	489.2	514.9	11.78	0.203
Carcass yield grade^1)^	2.55	2.58	0.17	0.905
Marbling score	6.23	6.17	0.44	0.927
Meat color	4.50	4.64	0.19	0.643
Fat color	3.00	3.00	0.05	1.000
Texture	1.00	1.36	0.08	0.080
Maturity	2.20	2.25	0.08	0.710
Quality grade	2.17	2.08	0.19	0.778
Frequencies of carcass yield grade (A:B:C, %)	7:29:64	6:31:63	-	-
Frequencies of carcass quality grade (1^++^:1^+^:1:2, %)	21:43:36:0	31:44:6:19	-	-

SSB, starch sugar by-product; SEM, standard error of the mean; LM, *Longissimus* muscles.

1) Carcass traits and grade were evaluated according to guidelines of the Animal Products Grading Service, South Korea (APGS, 1995).

**Table 6:** Effects of the dietary starch sugar by-product as a total mixed ration ingredient on longissimus muscle fatty acid composition in Hanwoo steers

Items	Control	SSB	SEM	p-value
C10:0	0.08	0.08	0.02	0.683
C12:0	0.09	0.10	0.02	0.567
C13:0	1.16	1.29	0.22	0.608
C14:0	3.17	3.73	0.51	0.284
C15:0	0.20	0.25	0.04	0.203
C16:0	26.31	29.27	1.16	0.052
C17:0	0.45	0.49	0.08	0.585
C18:0	9.89	10.07	0.71	0.863
C20:0	0.12	0.13	0.02	0.755
C21:0	0.04	0.04	0.00	0.753
C22:0	0.56	0.64	0.22	0.686
C23:0	0.02	0.02	0.00	0.180
C24:0	0.06	0.06	0.02	0.909
C14:1	0.95	1.25	0.25	0.341
C16:1	4.45	4.41	1.21	0.971
C17:1	0.53	0.50	0.07	0.646
C18:1n9-trans	0.11	0.11	0.01	0.877
C18:1n9-cis	47.40	43.07	1.19	0.055
C18:2n6-trans	0.23	0.23	0.04	0.959
C18:2n6-cis	3.29	3.37	0.63	0.898
C18:3n6	0.03	0.03	0.01	0.918
C18:3n3	0.08	0.08	0.01	0.377
C20:1	0.31	0.24	0.10	0.436
C20:2	0.27	0.33	0.10	0.521
C20:3n3	0.02	0.02	0.00	0.353
C20:4n6	0.02	0.02	0.00	0.490
C20:5n3	0.15	0.15	0.04	0.886
SFA	42.15	46.17	1.58	0.038
UFA	57.85	53.83	1.58	0.038
MUFA	53.75	49.59	0.67	0.079
PUFA	4.09	4.24	2.07	0.897
U/S	1.37	1.17	0.08	0.040

SSB, starch sugar by-product; SEM, standard error of the mean; SFA, saturated fatty acids; UFA, unsaturated fatty acids; MUFA, monounsaturated fatty acids; PUFA, polyunsaturated fatty acids; U/S, UFA/SFA.

## SUPPLEMENTARY MATERIAL

**Supplementary Table S1.** Physical and chemical characteristics of starch sugar by-product
