# Supplementary material for: Effects of feeding starch sugar by-products on in situ rumen disappearance rate, growth performance, and carcass characteristics of late finishing Hanwoo steers
Source: Anim Biosci. 2021 Aug 21;35(2):217–23. doi: 10.5713/ab.21.0126 (PMC8738940; doi:10.5713/ab.21.0126)
Supplement: Supplementary file 1 [file ab-21-0126-suppl.pdf]

**Supplementary Table S1.** Physical and chemical characteristics of starch sugar by-product[1] (n=10)

| Items | Mean | Median | SD | MIN | MAX | Skewness[2] | $2\times SE_S$[3] | Kurtosis[4] | $2\times SE_k$[5] |
|---|---|---|---|---|---|---|---|---|---|
| Physical characteristics | | | | | | | | | |
| Complex viscosity, hPa/s | 133.98 | 118.39 | 50.29 | 75.36 | 212.35 | 0.29 | 1.55 | -1.48 | 3.10 |
| Particle, size, μm | 557 | 570 | 128 | 289 | 703 | -1.16 | 1.55 | 1.07 | 3.10 |
| Density, DM $g/cm^3$ | 1.31 | 1.26 | 0.14 | 1.19 | 1.61 | 1.15 | 1.55 | 0.71 | 3.10 |
| Chemical compositions | | | | | | | | | |
| DM, % | 61.43 | 61.80 | 3.52 | 55.20 | 65.83 | -0.44 | 1.55 | -0.79 | 3.10 |
| CP, %DM | 14.43 | 13.00 | 7.63 | 6.01 | 33.46 | 1.93 | 1.55 | 4.57 | 3.10 |
| EE, %DM | 23.26 | 21.40 | 7.41 | 10.66 | 36.99 | 0.22 | 1.55 | 0.42 | 3.10 |
| NDF, %DM | 35.67 | 34.10 | 11.28 | 13.15 | 51.22 | -0.76 | 1.55 | 0.66 | 3.10 |
| ADF, %DM | 31.29 | 28.93 | 10.93 | 11.76 | 47.11 | -0.20 | 1.55 | -0.12 | 3.10 |
| Ash, %DM | 32.60 | 33.68 | 13.67 | 11.01 | 57.32 | 0.20 | 1.55 | -0.06 | 3.10 |
| WSC, %DM | 9.96 | 9.22 | 3.98 | 1.71 | 15.78 | -0.48 | 1.55 | 1.37 | 3.10 |
| GE, Kcal/kg | 4,198 | 3965 | 1,033 | 2,341 | 6,005 | 0.11 | 1.55 | 0.48 | 3.10 |
| pH | 4.14 | 3.71 | 0.80 | 3.41 | 5.33 | 0.97 | 1.55 | -1.22 | 3.10 |

[1]DM, dry matter; CP, crude protein; EE, ether extract; NDF, neutral detergent fiber; ADF, acid detergent fiber; WSC, water soluble carbohydrate; GE, gross energy; SD, Standard deviation; MIN, Minimum value in database, MAX, Maximum value in database.

[2]The degree of asymmetry of a distribution around its mean where $0 \pm 2 \times Se_S$ = normal.

[3]$SE_S$, square root (6/n).

[4]Characterizes the relative peakedness or flatness of a distribution, where $0 \pm 2 \times Se_k$ = normal.

[5]$SE_k$, square root (24/n)
